# Effects of paramylon‐rich *Euglena gracilis* EOD‐1 powder on visceral fat obesity in moderately obese Japanese adults: A randomized, double‐blind, placebo‐controlled, parallel‐group trial

**DOI:** 10.1002/fsn3.3130

**Published:** 2022-11-17

**Authors:** Seiichiro Aoe, Takanori Kawano, Junko Naito, Norihisa Nishida, Madoka Takahashi

**Affiliations:** ^1^ Department of Food Science, Faculty of Home Economics Otsuma Women's University Tokyo Japan; ^2^ Kobelco Eco‐Solutions Co., Ltd. Kobe Japan

**Keywords:** adiponectin, *Euglena gracilis*, HbA1c, paramylon, visceral fat area

## Abstract

To investigate whether supplementation of paramylon (PM)‐rich *Euglena gracilis* EOD‐1 powder (EOD‐1) reduces visceral fat obesity in moderately obese Japanese subjects. A randomized, double‐blind, placebo‐controlled intervention study was conducted involving 36 Japanese adults with a body mass index (BMI) ≥25 and <30 kg/m^2^. Subjects were randomly assigned into two groups to consume EOD‐1 capsules (EOD‐1 group, 2.6 g PM/day) or cellulose capsules (placebo group) for a 12‐week period. Anthropometric measurements including visceral fat area (VFA) and blood samples were measured at baseline and throughout the trial. There was no significant difference in VFA between the two groups, although subgroup analysis by gender showed a significant decrease in VFA in the male EOD‐1 group compared with the placebo group. Serum adiponectin levels in all subjects from the EOD‐1 group were significantly higher than in the placebo group. By comparison with the placebo group, the subjects in the EOD‐1 group showed a significant reduction in serum HbA1c levels. EOD‐1 intake led to a significant reduction in VFA in male subjects with moderate obesity (BMI 25–30 kg/m^2^). PM in EOD‐1 may contribute to preventing visceral fat obesity in male Japanese subjects. Moreover, PM may also contribute to improving glucose homeostasis in moderately obese Japanese adults.

## Graphical abstract

We studied whether paramylon‐rich *Euglena gracilis* EOD‐1 powder supplementation could improve visceral fat obesity. EOD‐1 was found to enhance VFA loss and increase serum adiponectin concentration in moderately obese male adult subjects after a 12‐week supplementation period (2.6 g/day PM). EOD‐1 may reduce the serum HbA1c concentration and HOMA‐R in both male and female subjects. Effect of PM EOD‐1 on visceral and subcutaneous fat areas (bars represent mean and SE). *: *p* < .05 (vs. baseline, paired *t*‐test), #: *p* < .05 (ANCOVA). □ baseline, ■ 12 weeks.

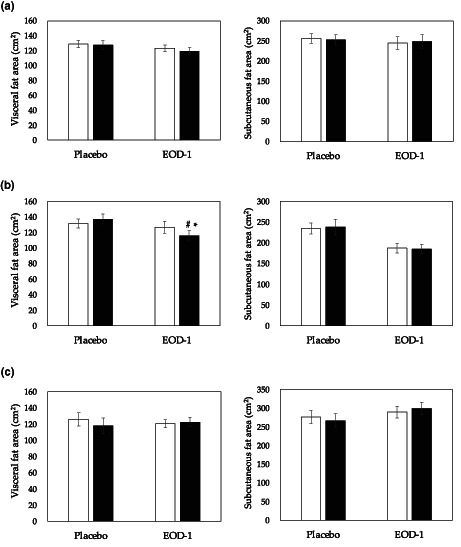

## 1 INTRODUCTION

Obesity is recognized as a major risk factor for morbidity and mortality, particularly lifestyle‐related disorders such as type 2 diabetes, hypertension, and dyslipidemia (Klop et al., 2013; Leitner et al., 2017; Natsis et al., 2020). Metabolic syndrome (MS) is a combination of medical conditions that increase the risk of coronary heart disease, diabetes, and stroke, including abdominal obesity, dyslipidemia, hypertension, and impaired glucose tolerance (Eckel et al., 2005). Adoption of a healthier diet, particularly increased consumption of dietary fiber, can markedly mitigate the symptoms associated with MS. Indeed, epidemiological results showed fiber in the diet was negatively correlated with the incidence of type 2 diabetes (InterAct Consortium, 2015; Yao et al., 2014), hypertension, dyslipidemia (Reynolds et al., 2019), and coronary heart disease (Wu et al., 2015). Unfortunately, however, the average Japanese diet contains relatively little fiber (<15 g/day; Ministry of Health, Labour and Welfare, Japan, 2018).

Generally, dietary fiber is classified according to its physicochemical properties such as water solubility and viscosity. Insoluble dietary fiber induces a significant increase in fecal volume, shortened intestinal transit time, and a substantial increase in stool frequency in healthy individuals due to its water‐holding capacity (Vuksan et al., 2008). Water‐soluble dietary fiber suppresses the digestion and absorption of carbohydrates and lipids, moderates postprandial blood glucose elevation, and normalizes serum cholesterol levels (Weickert & Pfeiffer, 2008). It is known that certain dietary fibers are metabolized by gut bacteria and the short‐chain fatty acids generated by this process affect energy and nutrient metabolism (Blaak et al., 2020; Koh et al., 2016). We previously showed that the intake of high β‐glucan barley by subjects with visceral fat obesity led to a significant reduction in VFA, body weight, BMI, and waist circumference (Aoe et al., 2017). It has been postulated that the visceral fat‐reducing effect of barley β‐glucan is due to the suppression of nutrient absorption due to its viscosity and the prebiotic action mediated by short‐chain fatty acids produced by intestinal microbiota (Aoe et al., 2020). However, β‐glucan classified as paramylon (PM) does not possess these properties (Aoe et al., 2019).

Paramylon is a starch‐like carbohydrate produced as a distinctive cellular deposit in *Euglena gracilis*. The structure of PM, which is classified as an insoluble dietary fiber, comprises β‐1,3‐glucan chains that adopt a triple helical arrangement (Marchessault & Deslamdes, 1979). Beta‐glucans are common in cereal grains, fungi, and algae, although their physicochemical properties vary considerably from source to source. Recent studies indicate the positive effects of PM supplements in terms of reducing fatigue and promoting immune function (Ishibashi et al., 2019; Kawano et al., 2020). However, to date, no human intervention study investigating the anti‐obesity effects of PM has been reported. We previously demonstrated that PM has beneficial effects on the prevention of obesity‐related parameters in animal experiments (Aoe et al., 2019). Specifically, dietary supplementation of PM was found to reduce abdominal fat accumulation, serum LDL‐cholesterol levels, and postprandial blood glucose rise. Moreover, the underlying mechanism was not mediated by the suppression of nutrient digestion and absorption or intestinal fermentation, but rather by modification of lipid metabolism‐related gene expression profiles in the liver (Aoe, Yamanaka, & Mio, 2021). Therefore, PM is considered to act differently to previously studied dietary fibers.

The purpose of this investigation was to confirm whether supplementation with PM‐rich *E. gracilis* EOD‐1 powder (EOD‐1) causes visceral fat area (VFA) depletion in moderately obese Japanese adults (BMI 25–30 kg/m^2^). Parameters related to glucose and lipid metabolism were also examined. The primary outcome was VFA, and the secondary outcomes were other anthropometric measurements and serum parameters related to metabolic syndrome. This is the first randomized, double‐blind, placebo‐controlled study designed to assess the anti‐obesity effect of PM in Japanese participants.

## 2 MATERIALS AND METHODS

### 2.1 Subjects

The study design conformed with regulations of the Institutional Review Board of Utsukushigaoka Hospital according to the ethical guidelines for medical and health research involving human subjects and the Declaration of Helsinki. All subjects gave written informed consent before participating in the study. The focus of this study was to examine the effects of EOD‐1 on abdominal fat reduction in adults with moderately elevated BMI.

The inclusion criteria were as follows:

1. Age 20–65 years
2. BMI 24–30 kg/m^2^
3. Waist circumference > 85 cm^2^ (male), >90 cm^2^ (female)
4. No drastic changes in lifestyle during the experimental period
5. No irregular eating habits or sleep patterns, for example, shift/night work
6. All subjects were given a detailed description of the study, which enabled them to fully understand the protocol and give written informed consent.

Individuals who had the following characteristics were excluded from the study:

1. Suffering from a malignant tumor, heart failure, myocardial infarction, or psychiatric disorder
2. Those being treated for a chronic disease
3. Individuals with a history of serious diseases (e.g., heart, liver, kidney, and digestive organs)
4. Excessive alcohol intake
5. Allergies (pharmaceutical/foods related to test supplements)
6. Individuals who had participated in other clinical trials within 3 months of giving informed consent
7. Individuals taking supplements containing β‐glucan
8. Individuals prescribed warfarin as medication
9. Those who were pregnant or plan to be pregnant, and those breastfeeding
10. Individuals with irregular eating habits or lifestyle rhythms, such as shift workers and late‐night workers
11. Individuals with severe anemia
12. Any male who donated 400 ml of whole blood 3 months prior to the start of the trial
13. Those suffering from an infectious disease
14. Judged by a medical doctor to be inappropriate for this study

Forty subjects were randomly assigned into each trial group (EOD‐1 group and placebo group) and then stratified by VFA, BMI, age, and sex. All subjects were Japanese men and women. The study was designed as a double‐blind, controlled, randomized trial. Subjects consumed five test capsules three times a day (15 capsules daily) at each meal for 12 weeks. The study protocol was registered at the University Hospital Medical Information Network Clinical Trials Registry (UMIN000043114).

### 2.2 Test supplements

The test powder of *E. gracilis* EOD‐1 rich in PM (79% PM) was obtained from Kobelco Eco‐Solutions Co., Ltd. Each test capsule and placebo capsule contained 175 mg of PM or cellulose, respectively. The placebo and test capsules looked identical.

### 2.3 Study design and intervention

Both subjects and researchers were unaware of the designation of capsules among the two groups. Subjects consumed five capsules (placebo or EOD‐1 capsules) three times a day (breakfast, lunch, and dinner) over a 12‐week period (i.e., 15 capsules/day). Individuals in the EOD‐1 group had a daily PM intake of 2.6 g (175 mg/capsule). Dosage of PM was chosen in accordance with previous reports that used test foods rich in barley β‐glucan (Aoe et al., 2017; Matsuoka et al., 2014). All subjects maintained their customary diets and abstained from excessive eating or drinking both prior to and during the experimental period. The study was conducted in Hokkaido (Japan) from March to May 2021. During the trial, subjects filled out daily capsule intake records. Moreover, each subject produced a three‐day record of their diet at baseline and during the final (12th) week of the trial. The intake records from this trial were analyzed using the Standard Tables of Food Composition in Japan 2020, eighth Revised Version (Ministry of Education, Culture, Sports, Science and Technology, 2020), and the software program EiyoPlus (Kenpakusha). Cases were excluded from the trial if the record showed a low daily consumption of capsules, or their recorded intake diary was not in compliance. Anthropometric measurements (body weight, height, body fat percentage, and VFA) were performed during the fourth, eighth, and final weeks of the trial. In all cases, blood samples were taken from a forearm vein.

### 2.4 Clinical analyses

CT scans were performed with a SOMATOM emotion 16 (Siemens Healthcare). The areas of visceral/subcutaneous fat were evaluated from individual slices. The body weight and height of each subject were determined, and the corresponding BMI calculated. Waist circumference (WC) was measured to the nearest 0.1 cm. Blood samples were obtained after an overnight fast using the services of a commercial laboratory (SAPPORO CLINICAL LABORATORY INC.). Serum insulin concentration was analyzed by chemiluminescent enzyme immunoassay (Lumipulse insulin‐N; Fujirebio Inc.). Serum leptin and adiponectin concentrations were determined using an enzyme‐linked immunosorbent assay (ELISA) kit (Quantikine® ELISA kit, R&D Systems). Serum IgA concentrations were also determined using a turbidimetric immunoassay (N‐Assay TIA IgA‐SH Nittobo; Nittobo Medical Co., Ltd.).

### 2.5 Statistical analyses

Sample sizes were based on a previous publication (Aoe, Yamanaka, Ohtoshi, et al., 2021). Specifically, >18 subjects were needed per group (type I error [α] = 0.05, 1–β = 0.80). Data normality was verified by means of a quantile–quantile plot. Homogeneity of variances was determined using Bartlett's test. All data were evaluated using a statistical software package IBM SPSS ver. 24.0 and 25.0 (IBM Japan Ltd.) and JMP (Version 16.0, SAS Institute Inc.). Change from baseline at each period between the placebo and EOD‐1 groups was analyzed by ANCOVA with the baseline as the covariate, if the two groups have equal slopes in the regression of each period data on baseline data. Data that were not applicable for ANCOVA were analyzed as follows. Variance between the placebo and EOD‐1 groups was evaluated by Student's *t*‐test or Wilcoxon rank‐sum test. Differences between baseline and each period data were analyzed by Dunnett's multiple comparison test with a block factor as subjects, Steel's multiple comparison test, or paired *t*‐test (baseline vs. 12 weeks only). Where gender differences were evident, measurements were analyzed after stratification by gender. In all analyses, a two‐sided *p*‐value <.05 was considered significant.

## 3 RESULTS

### 3.1 Subject characteristics

**FIGURE 1:**
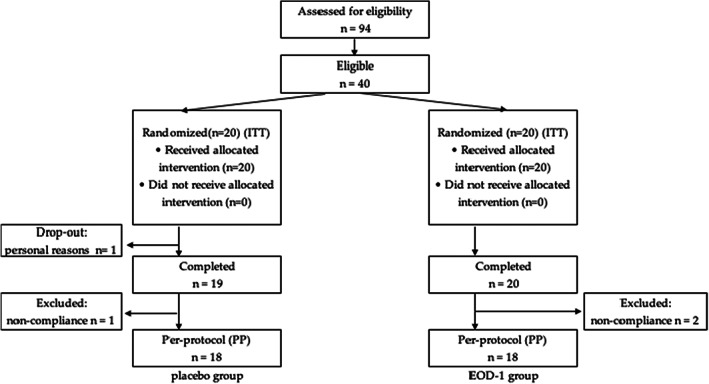
Flow diagram of subjects throughout the 12‐week intervention study (ITT, Intention to treat).

In all, 40 subjects were found to be eligible to participate in this trial. During the course of the experimental period, one subject withdrew from the study for personal reasons. All other subjects completed the trial. A total of four subjects were excluded from the trial: One subject from the placebo group withdrew for personal reasons, and the other three were excluded for noncompliance (one from the placebo and two from the EOD‐1 group, see Figure 1). Table 1 summarizes the baseline attributes of the subjects. The two groups were similar in age, body weight, and height. Significant changes from the baseline were detected for several parameters. However, no abnormal or adverse changes in blood parameters were observed between the two groups during the intervention period (Table S1). The intake rate of the test supplement was 100% for 36 subjects and 99.6% for the remainder. All of these subjects were included in the data analysis. No adverse events related to the intake of EOD‐1 capsules (e.g., gastrointestinal issues) were found during the trial period. Statistical analyses were carried out for the subjects in the placebo group (*n* = 18) and EOD‐1 (*n* = 18) groups in the per‐protocol set analysis.

**TABLE 1:** Baseline characteristics of subjects

Group	n	Male	Female	Age (years)	Height (cm)	Body weight (kg)
Placebo	18	9	9	51.0 ± 2.0	165.6 ± 2.0	76.6 ± 2.3
EOD‐1	18	8	10	49.6 ± 2.8	165.2 ± 2.0	73.4 ± 1.8

*Note*: Mean ± standard error (SE). There were no statistically significant differences between the two groups.

### 3.2 Energy and nutrient intake

Table 2 outlines the energy and nutrient intake of subjects during the trial. There was no significant difference in calorie or nutrient (protein, fat, carbohydrate, cholesterol, and total dietary fiber) intake between the placebo and EOD‐1 groups immediately prior to and during the experimental period.

**TABLE 2:** Daily energy and nutrient intake during the trial

Week	0	12	12–0 W	*p* (12–0 W)^†^
Energy (kcal)				
Placebo	1922 ± 70	1827 ± 87	−95 ± 52	.22
EOD‐1	1900 ± 64	1945 ± 121	45 ± 99	
Protein (g)				
Placebo	73.4 ± 2.6	70.1 ± 3.8	−3.1 ± 2.8	.44
EOD‐1	75.9 ± 3.2	76.4 ± 4.4	0.5 ± 3.8	
Fat (g)				
Placebo	65.6 ± 3.4	60.4 ± 3.8	−5.1 ± 2.9	.69
EOD‐1	67.2 ± 3.7	64.0 ± 5.5	−3.1 ± 3.9	
Carbohydrate (g)				
Placebo	243.8 ± 11.4	233.7 ± 12.9	−10.1 ± 9.1	.15
EOD‐1	236.5 ± 10.8	254.2 ± 19.4	17.7 ± 16.5	
Cholesterol (mg)				
Placebo	373.3 ± 23.4	345.1 ± 22.0	−27.1 ± 16.6	.37
EOD‐1	362.4 ± 24.0	363.0 ± 28.6	0.7 ± 26.1	
Total dietary fiber (g)				
Placebo	13.6 ± 1.5	11.6 ± 0.9	1.1 ± 3.3	.84
EOD‐1	13.6 ± 1.2	12.6 ± 1.1	−0.2 ± 5.2	

*Note*: Mean ± standard error (SE). Average daily record for 3 days.

^†^ *p* values by Student's *t*‐test.

### 3.3 Anthropometric measurements and visceral and subcutaneous fat areas

**FIGURE 2:**
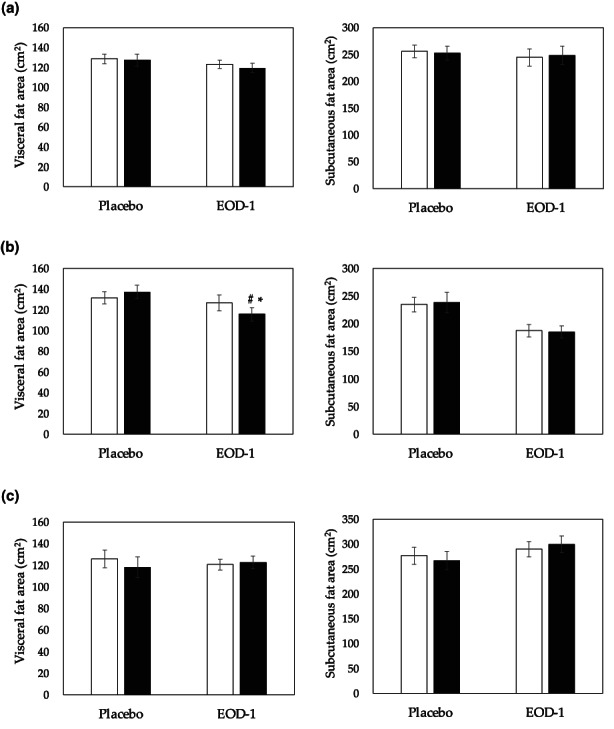
Effect of PM EOD‐1 on visceral and subcutaneous fat areas (bars represent means and SE): (a) overall (male and female), (b) male, and (c) female. *: *p* < .05 (vs. baseline, paired *t*‐test), #: *p* < .05 (ANCOVA). □ baseline, ■ 12 weeks

Alterations in body weight, waist circumference, and body fat percentage after 12‐week consumption of EOD‐1 are shown in Table 3. Representative examples of computed tomography (CT)‐scans used to determine area‐based, densitometric quantification of adipose tissue comprising visceral fat as well as subcutaneous fat area at baseline and after 12 weeks are shown in Figure S1. Estimations of visceral and subcutaneous fat areas are given in Figure 2. There was no significant difference in VFA between the two groups. However, the data were also evaluated after stratification by gender. In males, the VFA was significantly reduced in the EOD‐1 compared with the placebo group by ANCOVA. Waist circumference for overall and female subjects at 4 weeks and male subjects at 8 and 12 weeks was significantly reduced compared with baseline in the EOD‐1 group. No significant differences between the placebo group and the EOD‐1 in body weight, body fat percentage, and subcutaneous fat area were observed in males. In females, no significant changes were observed in any anthropometric measurements and fat area except for a transitory reduction of body weight at 8 weeks in the placebo group and waist circumference at 4 weeks in the EOD‐1 group.

**TABLE 3:** Effect of PM EOD‐1 on body weight, waist circumference, and body fat percentage

	Group	Baseline	4 weeks	8 weeks	12 weeks	ANCOVA
Overall (male and female)						
Body weight (kg)	Placebo	76.6 ± 2.3	76.5 ± 2.4	76.0 ± 2.5	76.4 ± 2.5	0.828
	EOD‐1	73.4 ± 1.8	73.0 ± 1.7	73.1 ± 1.8	72.9 ± 1.8	
Waist circumference (cm)	Placebo	96.7 ± 1.0	95.4 ± 2.0	96.0 ± 1.2	94.8 ± 1.2	0.706
	EOD‐1	95.8 ± 1.1	92.6 ± 1.2*	93.8 ± 1.5	93.5 ± 1.6	
Body fat percentage (%)	Placebo	34.6 ± 1.4	34.5 ± 1.3	34.3 ± 1.3	34.4 ± 1.4	0.717
	EOD‐1	34.4 ± 1.8	34.2 ± 1.9	34.7 ± 1.9	34.5 ± 1.9	
Male						
Body weight (kg)	Placebo	83.2 ± 2.9	83.4 ± 3.1	83.3 ± 3.1	83.8 ± 3.2	0.371
	EOD‐1	77.9 ± 2.8	77.4 ± 2.7	77.6 ± 3.0	77.2 ± 3.0	
Waist circumference (cm)	Placebo	97.8 ± 1.8	97.2 ± 1.9	96.5 ± 2.0	96.1 ± 2.1	0.114
	EOD‐1	92.9 ± 1.1	90.5 ± 1.6	89.3 ± 1.3*	88.4 ± 1.4*	
Body fat percentage (%)	Placebo	30.2 ± 1.5	30.3 ± 1.4	30.1 ± 1.4	30.3 ± 1.6	0.794
	EOD‐1	26.5 ± 0.9	26.2 ± 0.9	26.8 ± 0.9	26.5 ± 1.2	
Female						
Body weight (kg)	Placebo	70.0 ± 1.7	69.5 ± 1.7	68.6 ± 1.7*	69.1 ± 1.8	0.646
	EOD‐1	69.8 ± 1.5	69.5 ± 1.5	69.5 ± 1.6	69.5 ± 1.6	
Waist circumference (cm)	Placebo	95.5 ± 0.9	93.5 ± 0.8	95.4 ± 1.3	93.5 ± 1.0	n.a.
	EOD‐1	98.0 ± 1.4	94.3 ± 1.7*	97.4 ± 1.8	97.6 ± 1.7	
Body fat percentage (%)	Placebo	38.9 ± 1.0	38.6 ± 0.9	38.5 ± 0.9	38.5 ± 1.2	0.422
	EOD‐1	40.7 ± 1.1	40.6 ± 1.1	41.0 ± 1.1	40.8 ± 1.0	

*Note*: Mean ± standard error (SE).

* *p* < .05 (vs. baseline, Dunnett's test), n.a., not applicable for ANCOVA.

### 3.4 Serum lipid and other parameters

Serum lipid values are given in Table 4. No significant differences were identified in serum lipid levels. Serum total cholesterol and LDL‐cholesterol concentrations were significantly higher in the EOD‐1 group than in the placebo group by Student's *t*‐test at 0, 8, and 12 weeks and 0, 4, and 12 weeks, respectively (Table S1).

**TABLE 4:** Effects of PM EOD‐1 on serum lipids

	Group	Baseline	4 weeks	8 weeks	12 weeks	ANCOVA
Triglyceride (mmol/L)	Placebo	1.77 ± 0.20	2.01 ± 0.31	1.72 ± 0.20	1.75 ± 0.23	0.890
	EOD‐1	1.99 ± 0.25	2.00 ± 0.25	2.46 ± 0.57	1.83 ± 0.19	
HDL‐cholesterol (mmol/L)	Placebo	1.59 ± 0.07	1.53 ± 0.09	1.61 ± 0.09	1.54 ± 0.08	0.916
	EOD‐1	1.60 ± 0.10	1.56 ± 0.09	1.55 ± 0.09	1.54 ± 0.09	
LDL‐cholesterol (mmol/L)	Placebo	3.94 ± 0.16	3.69 ± 0.16	3.84 ± 0.17	3.84 ± 0.18	0.320
	EOD‐1	4.35 ± 0.23	4.18 ± 0.22	4.18 ± 0.25	4.34 ± 0.22	
Total cholesterol (mmol/L)	Placebo	6.33 ± 0.20	6.04 ± 0.19	6.17 ± 0.20	6.05 ± 0.21	0.319
	EOD‐1	6.80 ± 0.23	6.54 ± 0.22	6.76 ± 0.24	6.62 ± 0.22	

*Note*: Mean ± standard error (SE).

**FIGURE 3:**
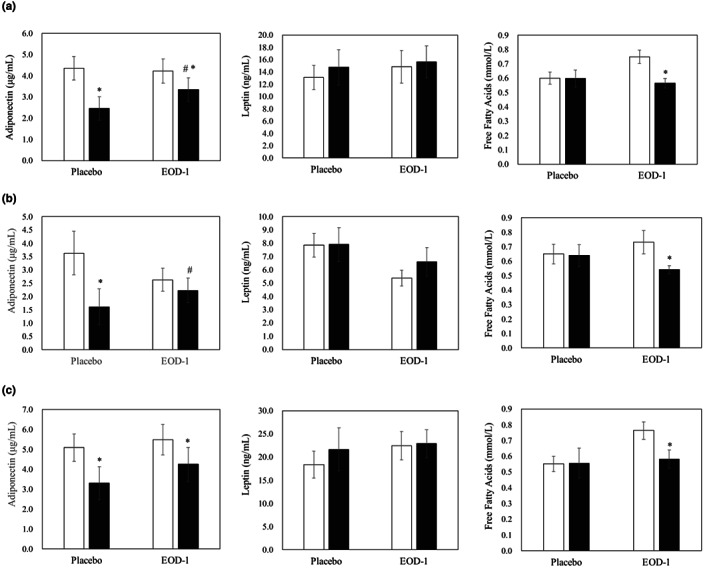
Effect of PM EOD‐1 on serum adipocytokine and free fatty acid concentration (bars represent means and SE): (a) overall (male and female), (b) male, and (c) female. *: *p* < .05 (vs. baseline, paired *t*‐test), #: *p* < .05 (ANCOVA). □ baseline, ■ 12 weeks

Serum adipocytokine and free fatty acid concentrations are shown in Figure 3. The serum adiponectin concentrations were significantly higher in the overall and male EOD‐1 groups than in the placebo group by ANCOVA, but reduced in the male placebo group and in both female groups compared with baseline. A significant difference in serum leptin concentration was not observed between the placebo and EOD‐1 groups. Serum‐free fatty acid concentrations were significantly lower in the EOD‐1 group than in baseline.

Parameters related to glucose homeostasis are shown in Table 5. Serum fasting blood glucose was increased in the EOD‐1 group compared with baseline. Overall (male and female), HbA1c levels in the EOD‐1 group were significantly lower than in the placebo group by ANCOVA. The same trend was observed for both male and female (*p* = .072 and .061, respectively) by ANCOVA. A particularly significant reduction in HbA1c was observed at 12 weeks versus baseline for males and females in the EOD‐1 group. Serum insulin levels in the male EOD‐1 group were significantly lower than the male placebo group by ANCOVA. HOMA‐R calculated from fasting blood glucose and insulin, which is an insulin resistance marker, in the male EOD‐1 group was significantly lower than in the male placebo group by ANCOVA. Serum total protein concentrations were significantly higher at 4, 8, and 12 weeks for the EOD‐1 groups than in the placebo group by Student's *t*‐test (Table S1). However, the values in both groups were within the normal range.

**TABLE 5:** Effect of PM EOD‐1 on glucose homeostasis

	Group	Baseline	4 weeks	8 weeks	12 weeks	ANCOVA
Overall (male and female)						
Fasting blood glucose (mmol/L)	Placebo	49.5 ± 0.7	49.5 ± 0.7	50.9 ± 0.7	50.8 ± 0.8	0.244
	EOD‐1	48.1 ± 1.1	50.4 ± 1.4*	50.2 ± 1.3*	50.6 ± 1.2*	
HbA1c (%)	Placebo	5.58 ± 0.06	5.55 ± 0.06	5.55 ± 0.06	5.50 ± 0.05	0.007
	EOD‐1	5.40 ± 0.07	5.38 ± 0.08	5.37 ± 0.08	5.26 ± 0.06*	
Insulin (pmol/L)	Placebo	52.7 ± 4.7	50.7 ± 5.2	56.7 ± 5.9	59.5 ± 5.2	0.528
	EOD‐1	53.5 ± 4.6	59.9 ± 7.1	53.9 ± 6.0	55.9 ± 7.3	
HOMA‐R	Placebo	1.63 ± 0.16	1.57 ± 0.17	1.79 ± 0.19	1.89 ± 0.18	0.744
	EOD‐1	1.60 ± 0.14	1.89 ± 0.23	1.69 ± 0.20	1.78 ± 0.25	
Male						
Fasting blood glucose (mmol/L)	Placebo	49.8 ± 1.2	50.5 ± 1.2	51.2 ± 1.3	52.1 ± 1.3*	0.739
	EOD‐1	49.2 ± 1.8	52.1 ± 2.7*	52.0 ± 2.4*	51.1 ± 2.1	
HbA1c (%)	Placebo	5.46 ± 0.08	5.44 ± 0.08	5.47 ± 0.10	5.40 ± 0.07	0.072
	EOD‐1	5.44 ± 0.14	5.38 ± 0.16	5.40 ± 0.15	5.30 ± 0.10*	
Insulin (pmol/L)	Placebo	52.1 ± 7.0	51.1 ± 8.0	58.4 ± 10.4	64.0 ± 8.8	0.011
	EOD‐1	51.7 ± 7.4	54.4 ± 9.0	50.5 ± 6.1	48.0 ± 8.2	
HOMA‐R	Placebo	1.62 ± 0.23	1.62 ± 0.27	1.86 ± 0.33	2.09 ± 0.31	0.030
	EOD‐1	1.58 ± 0.24	1.78 ± 0.31	1.64 ± 0.23	1.55 ± 0.31	
Female						
Fasting blood glucose (mmol/L)	Placebo	49.2 ± 0.9	48.5 ± 0.8	50.6 ± 0.8	49.5 ± 0.8	n.a.
	EOD‐1	47.3 ± 1.5	49.0 ± 1.4	48.8 ± 1.2	50.2 ± 1.5*	
HbA1c (%)	Placebo	5.71 ± 0.09	5.66 ± 0.08	5.63 ± 0.06	5.60 ± 0.06	0.061
	EOD‐1	5.37 ± 0.08	5.38 ± 0.09	5.34 ± 0.09	5.23 ± 0.08*	
Insulin (pmol/L)	Placebo	53.3 ± 6.6	50.3 ± 7.2	55.1 ± 6.4	54.9 ± 5.6	0.619
	EOD‐1	55.0 ± 6.0	64.2 ± 10.8	56.5 ± 9.9	62.2 ± 11.4	
HOMA‐R	Placebo	1.65 ± 0.23	1.53 ± 0.23	1.73 ± 0.21	1.70 ± 0.19	0.463
	EOD‐1	1.61 ± 0.18	1.98 ± 0.35	1.74 ± 0.32	1.97 ± 0.38	

*Note*: Mean ± standard error (SE).

* *p* < .05 (vs. baseline, Dunnett's test), n.a., not applicable for ANCOVA.

For additional analysis, serum IgA concentrations, as an immunological marker related to obesity, are shown in Table S2. An overall significant increase in serum IgA concentration was seen in the EOD‐1 group compared with the placebo group by ANCOVA. Similar analysis showed the male EOD‐1 group had a significantly higher serum IgA concentration over the male‐specific placebo group. Serum uric acid concentrations (moderately high normal range subjects, 327–476 μmol/L, *n* = 5 (EOD‐1), *n* = 6 (placebo)) in the male EOD‐1 subgroup were significantly lower than in the placebo subgroup (Figure S2).

## 4 DISCUSSION

Here, a parallel, double‐blind, placebo‐controlled trial was conducted to investigate the effect of dietary supplementation of PM on visceral fat obesity in Japanese adults. We randomly allotted 36 moderately obese (BMI 25–30 kg/m^2^) subjects into two groups: a placebo supplement or a PM EOD‐1 (2.6 g PM/day) group. For male subjects, there was a significant VFA loss within the EOD‐1 group compared with the placebo group. Moreover, the serum adiponectin concentration was significantly higher at 12 weeks in the EOD‐1 group than in the test group.

We initially hypothesized that supplementation with an EOD‐1 might enhance VFA loss and improve metabolic parameters in Japanese subjects with moderate obesity (BMI 25–30 kg/m^2^). This study represents the first randomized, double‐blind, placebo‐controlled trial to evaluate the effects of PM EOD‐1. Based on the results of our animal study (Aoe, Yamanaka, & Mio, 2021), the observed decrease in VFA is likely to be mediated by the activation of PPARα in the liver. PM is a type of insoluble dietary fiber (β‐glucan) that does not affect the digestion and absorption of lipids and is not metabolized by intestinal bacteria. Dietary fiber has been reported to bring about a reduction in food intake by prolonging gastric residence time, thereby enhancing satiety (Kristensen & Jensen, 2011). However, because PM forms insoluble particles that do not absorb water, it is thought to have no effect on gastric emptying time.

Currently, it is believed that PM may act by inhibiting the transport of fatty acids in the intestinal tract with a resulting suppression in chylomicron secretion (Aoe, Yamanaka, & Mio, 2021). Correspondingly, the decrease in the secretion of chylomicrons may suppress the accumulation of fat within adipose tissue. It has been reported that adiponectin secretion decreases with increasing fat accumulation (Gariballa et al., 2019). Given that adiponectin activates PPARα, this pathway is considered to be a candidate for the mechanism (Yadav et al., 2013).

In this study, a decrease in VFA after ingestion of EOD‐1 was seen specifically in male subjects. The development of obesity and cardiovascular disease differs greatly between men and women (Odunsi et al., 2010). In general, men have a higher tendency to accumulate visceral adipose tissue by comparison with women. By contrast, women tend to increase fat mass in proportion to their body weight due to increased subcutaneous adipose tissue. The incidence of MS for Japanese women is much lower than for men (14.3% for women but 25% for men; Link & Reue, 2017). Our previous study investigated kelp powder intake, which correlated with a reduction in body fat percentage specifically in male subjects (Aoe, Yamanaka, Ohtoshi, et al., 2021). Further studies regarding gender differences are needed to elucidate the effect of EOD‐1 intake on visceral fat reduction in subjects with abdominal obesity.

Serum adiponectin concentrations decreased in both groups at the end of the study. An association between insufficient vitamin D and lower circulating adiponectin in subjects with abnormal glucose tolerance has been reported (Nimitphong et al., 2009). This intervention trial was conducted at the end of the winter season in Hokkaido. Long‐term inadequate sun exposure may have reduced serum vitamin D status and decreased overall serum adiponectin levels. Another report showed that cold exposure increased adiponectin levels in men (Imbeault et al., 2009). The effects of seasonal variation on serum adipocytokines should be examined in a future study. However, the EOD‐1 group had significantly higher serum adiponectin concentrations over the placebo group at 12 weeks. This observation might arise from the suppression of visceral fat accumulation in the EOD‐1 group. Adiponectin also improves glucose tolerance through the activation of AMP kinase (Yamauchi et al., 2002). In this study, higher adiponectin levels caused a reduction in HbA1c levels. It is thought that an improvement in glucose tolerance by EOD‐1 intake may be directly related to the effect mediated by adiponectin. Reduction in the serum‐free fatty acid concentration is also reported to improve glucose intolerance (Jung & Bu, 2020). Thus, changes in the levels of adiponectin and free fatty acid in serum might be a key factor in reducing the concentrations of HbA1c and HOMA‐R.

It has been reported that EOD‐1 reduces fatigue by improving antioxidant potential (Kawano et al., 2020). PM has been reported to reduce oxidative damage to cells, improve oxidatively induced liver damage in rats, and increase liver reductase activity (Sugiyama et al., 2009). Proinflammatory status is considered to be a risk factor for MS (Eckel et al., 2005). Thus, the anti‐obesity effect of PM might arise from its antioxidative action, although the molecular mechanism remains unclear.

Serum IgA levels were increased following EOD‐1 intake, which was consistent with the findings of a previous report (Aoe et al., 2019). PM has an activating effect on intestinal epithelial cells and may stimulate the secretion of slgA (Spaeth et al., 1994). Human studies investigating dietary supplementation of PM‐rich EOD‐1 powder identified the production of PM‐specific IgA antibodies and enhanced salivary IgA antibody titers (Ishibashi et al., 2019). Data from the present study are consistent with these previous reports.

Our previous animal experiments showed that the intake of PM led to lower serum LDL‐cholesterol concentrations (Aoe et al., 2019). However, no such observation was made in the present study. Serum LDL‐cholesterol levels were not used for stratification during the randomization of subjects into the placebo and test groups. The levels of LDL‐cholesterol in the EOD‐1 group were subsequently found to be significantly higher than in the placebo group at baseline. Inconsistent serum cholesterol levels between the two groups at baseline may explain why no significant difference could be detected. Further human studies are needed to examine the effect of PM on cholesterol metabolism.

A major limitation of the present study was the small sample size. As such, a larger follow‐up study is planned to elucidate the effect of PM on VFA loss.

In conclusion, EOD‐1 was found to enhance VFA loss and increase serum adiponectin concentration in moderately obese male adult subjects after a 12‐week supplementation period (2.6 g/day PM). In addition, EOD‐1 may reduce the serum HbA1c concentration and HOMA‐R in both male and female subjects.

## FUNDING INFORMATION

This study was financially supported by Kobelco Eco‐Solutions Co., Ltd. (Kobe, Japan).

## CONFLICT OF INTEREST

Four of the authors (T.K., J.N., N.N., and M.T.) are salaried employees of Kobelco Eco‐Solutions Co., Ltd. The remaining author (S.A.) has no conflict of interest to disclose. The PM used in this study was provided by Kobelco Eco‐Solutions Co., Ltd.

## Supporting information


Appendix S1

## Data Availability

Research data are not shared.
