# Supplementary material for: Effects of paramylon‐rich Euglena gracilis EOD‐1 powder on visceral fat obesity in moderately obese Japanese adults: A randomized, double‐blind, placebo‐controlled, parallel‐group trial
Source: Food Sci Nutr. 2022 Nov 17;11(2):953–62. doi: 10.1002/fsn3.3130 (PMC9922139; doi:10.1002/fsn3.3130)
Supplement: Supplementary file 1 — Appendix S1 [file FSN3-11-953-s001.docx]

**Supplementary Materials**

**Title:** Effects of Paramylon rich *Euglena Gracilis* EOD-1 Powder on Visceral Fat Obesity in Moderately Obese Japanese Adults: A Randomized, Double-Blind, Placebo-Controlled, Parallel-Group Trial

**Supplemental Table 1** Serum biochemistry values (ITT)

* : p<0.05 (vs Placebo, Student's t-test) #: p<0.05 (vs 0w, Paired t-test) Mean ± standard error (SE)

**Supplemental Table 2** Serum IgA concentration

| **IgA (g/L)** | **Group** | **Baseline** | **4 weeks** | **8 weeks** | **12 weeks** | **ANCOVA** |
| --- | --- | --- | --- | --- | --- | --- |
| Overall | Placebo | 2.41±0.22 | 2.30±0.21* | 2.31±0.21* | 2.26±0.21* | 0.048 |
|  | EOD-1 | 2.62±0.17 | 2.55±0.17 | 2.59±0.17 | 2.55±0.17 |  |
|  |  |  |  |  |  |  |
| Male | Placebo | 2.65±0.32 | 2.50±0.30 * | 2.49±0.29 * | 2.45±0.29 * | 0.006 |
|  | EOD-1 | 2.77±0.21 | 2.73±0.22 | 2.81±0.21 | 2.77±0.21 |  |
|  |  |  |  |  |  |  |
| Female | Placebo | 2.17±0.30 | 2.11±0.30 | 2.13±0.32 | 2.07±0.30 * | 0.644 |
|  | EOD-1 | 2.51±0.26 | 2.41±0.24 | 2.42±0.25 | 2.37±0.25 * |  |

*: p<0.05（vs baseline, Dunnett’s test）. Mean ± standard error (SE)


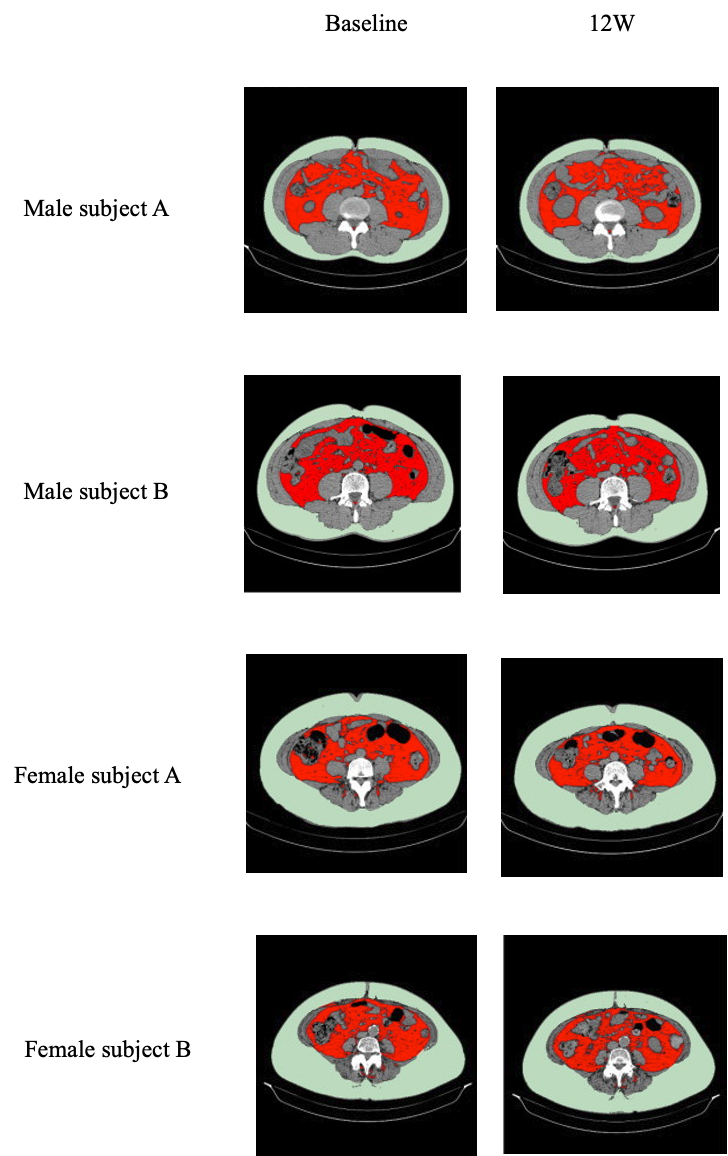


**Supplemental Figure 1** Representative examples of computed tomography (CT)-scans with area-based, densitometric quantificationof adipose tissue containing visceral fat (red) and subcutaneous fat area (green) in EOD-1 supplemented subjects at baseline and after the 12 week trial period (12W).


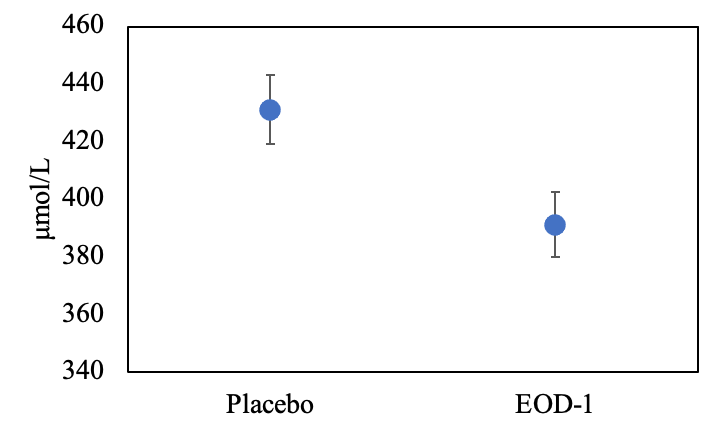


#

**Supplemental Figure 2** Serum uric acid concentration in the male subgroup (327-476μmol/L)

#p<0.05 (ANCOVA). Bars represent standard error (n=6; Placebo, n=5; EOD-1)
